# Association between genetic polymorphisms of NRF2, KEAP1, MAFF, MAFK and anti-tuberculosis drug-induced liver injury: a nested case-control study

**DOI:** 10.1038/s41598-019-50706-y

**Published:** 2019-10-04

**Authors:** Shixian Chen, Hongqiu Pan, Yongzhong Chen, Lihuan Lu, Xiaomin He, Hongbo Chen, Ru Chen, Siyan Zhan, Shaowen Tang

**Affiliations:** 1 Department of Epidemiology, School of Public Health, Nanjing Medical University, Nanjing, 211166 China; 2 Department of tuberculosis, The third people’s hospital of Zhenjiang affiliated to Jiangsu University, Zhenjiang, 212005 China; 3 Department of tuberculosis, The second people’s hospital of Changshu, Changshu, 215500 China; 4 Department of infectious Disease, The people’s hospital of Taixing, Taixing, 225400 China; 5 Department of infectious Disease, The people’s hospital of Jurong, Jurong, 212400 China; 6 Department of Epidemiology and Biostatistics, School of Public Health, Peking University Health Science Centre, Beijing, 100191 China

**Keywords:** Molecular medicine, Risk factors

## Abstract

Reactive metabolites of anti-tuberculosis (anti-TB) drugs can result in excessive reactive oxygen species (ROS), which are responsible for drug-induced liver injury. The nuclear factor erythroid 2-related factor 2 (Nrf2) - antioxidant response elements (ARE) (Nrf2-ARE) signaling pathway plays a crucial role in protecting liver cells from ROS, inducing enzymes such as phase II metabolizing enzymes and antioxidant enzymes. Based on a Chinese anti-TB treatment cohort, a nested case-control study was performed to explore the association between 13 tag single-nucleotide polymorphisms (tagSNPs) in the *NRF2*, *KEAP1*, *MAFF*, *MAFK* genes in Nrf2-ARE signaling pathway and the risk of anti-TB drug-induced liver injury (ATLI) in 314 cases and 628 controls. Conditional logistic regression models were used to calculate odds ratios (ORs) and 95% confidence intervals (CIs) after adjusting weight and usage of hepatoprotectant. Patients carrying the TC genotype at rs4243387 or haplotype C-C (rs2001350-rs6726395) in *NRF2* were at an increased risk of ATLI (adjusted OR = 1.362, 95% CI: 1.017–1.824, P = 0.038; adjusted OR = 2.503, 95% CI: 1.273–4.921, P = 0.008, respectively), whereas patients carrying TC genotype at rs2267373 or haplotype C-G-C (rs2267373-rs4444637-rs4821767) in *MAFF* were at a reduced risk of ATLI (adjusted OR = 0.712, 95% CI: 0.532–0.953, P = 0.022; adjusted OR = 0.753, 95% CI: 0.587–0.965, P = 0.025, respectively). Subgroup analysis also detected a significant association between multiple tagSNPs (rs4821767 and rs4444637 in *MAFF*, rs4720833 in *MAFK*) and specific clinical patterns of liver injury under different genetic models. This study shows that genetic polymorphisms of *NRF2*, *MAFF* and *MAFK* may contribute to the susceptibility to ATLI in the Chinese anti-TB treatment population.

## Introduction

Tuberculosis (TB) has existed throughout human history and remains a serious public health conce^1^. In 2017, an estimated 10.0 million people fell ill with TB and about 1.3 million patients died^1^. The Directly Observed Treatment Short-course (DOTS), an international recommendation strategy, remains the cornerstone of TB control in developing countries. TB can be treated by taking several anti-TB drugs for a minimum of 6 months: daily oral doses with a combination of rifampicin (RIF/R), isoniazid (INH/H), pyrazinamide (PZA/Z), and ethambutol (EMB/E) for 2 months, followed by 4 months of RIF and INH^2^. However, many studies have shown that utilization of multidrug regimens can cause unbearable adverse drug reactions (ADRs), such as gastrointestinal disorders, hepatotoxicity, allergic reactions, arthralgia, neurological disorders and so on^3^. These ADRs lead to non-adherence and treatment interruption, and they contribute to eventual treatment failure, relapse or the emergence of drug-resistance^4^. Among these ADRs, the most serious adverse reaction is anti-TB drug-induced liver injury (ATLI), which is often fatal^5^.

The pathophysiology of ATLI is still unclear^5^. However, most studies show that the development of ATLI is a complicated process related to drug, host and genetic susceptibility^6^. The experimental and clinical research indicates that reactive metabolites, rather than direct toxicity of the anti-TB drugs, are responsible for ATLI^7^, which occurs when these metabolites irreversibly bind to and modify many cellular components, particularly enzymes, and induce the production of excessive reactive oxygen species (ROS)^6,8^. Furthermore, ROS induce lipid peroxidation and cell death^6^. For example, recent research showed ROS accumulation and apoptosis could be induced by INH in HepG2 as well as THLE-2 cells^9^. Oxidative stress and beyond may contribute to the hepatic toxicity induced by first-line anti-TB drugs^10^. Liver cells can neutralize the extra ROS by antioxidant activities involving a variety of non-enzymatic and enzymatic mechanisms, such as glutathione S-transferases, NAD(P)H: quinone oxidoreductase, and glutamate-cysteine ligase^11,12^. Zhang *et al*. observed that the level of antioxidant enzymes and non-enzymatic antioxidants (superoxide dismutase, total antioxidant capacity, glutathione and malondialdehyde (MDA)) were changed in PZA-treated Wistar rats^13^. Additionally, in young rats, INH-RIF can directly increase ROS and consume glutathione, damaging the hepatic cell^14^. In a population-based study, the activity of glutathione was reduced and the level of MDA was increased in an ATLI group^15^. All these studies suggest that the accumulation of ROS in the liver is a potential mechanism of drug-induced liver injury^16^. Numerous mammalian studies have shown that the nuclear factor erythroid 2-related factor 2 (Nrf2) signaling molecules, activated by ROS, play an important role in transcriptional activation of downstream genes such as antioxidant and detoxification genes^17^.

Nrf2, a transcription factor that resists oxidative stress, belongs to the Cap-n-collar (CNC) basic leucine zipper family^18^. Under normal conditions, Nrf2 combines with kelch-like ECH associating protein 1 (Keap1) in the cytosol, which results in the activity of Nrf2 being temporarily inhibited. Upon exposure to oxidative stress or electrophilic, Nrf2 is released from Keap1 translocates to the nucleus, where it heterodimerizes with one of the small musculoaponeurotic fibrosarcoma (sMaf) proteins^17^. The highly homologous sMafs, MafF, MafK and MafG, are localized predominantly in the nucleus and previous studies have linked their functions, by virtue of their heterodimerization with the CNC family of transcription factors, to the stress response and detoxification pathways^19^. Heterodimers of Nrf2 and sMaf bind to antioxidant response elements (AREs) that boost the expression and transcription of phase II metabolizing enzymes and antioxidant proteins^20^. So, liver tissue can scavenge ROS by phase II metabolizing enzymes and antioxidant enzymes to keep oxidation and antioxidant balance, and the expression of these enzymes is mediated by the Nrf2-ARE signaling pathway.

In the Nrf2-ARE signaling pathway, it is the first and the crucial step that Nrf2 detaches from Keap1 in cytoplasm, moves to heterodimerizes with sMaf in nucleus. This process involves translocation of some relevant genes, such as *NRF2*, *KEAP1*, *MAFF*, *MAFK* and *MAFG* gene. It is reasonable to speculate that genetic variation in these genes may affect signal transduction during oxidative stress, resulting in ROS not being cleared in a timely fashion. So, in present study, we hypothesized that the genetic polymorphisms in the Nrf2-ARE signaling pathway may play an important role in susceptibility to ATLI. To test this hypothesis, 13 tag single-nucleotide polymorphisms (tagSNPs) in *NRF2*, *KEAP1*, *MAFF*, *MAFK* genes were analyzed to determine the role of tagSNPs in Chinese ATLI patients.

## Results

### Demographical and clinical data

Between April 2014 and December 2016, 3046 newly diagnosed TB patients were initially identified from hospitals, and 2209 patients finished the anti-TB treatment. A total of 314 ATLI cases and 628 non-ATLI controls was included in present study from the cohort. Among the 314 ATLI cases, 150 patients (47.8%) had a hepatocellular type; 23 patients (7.3%) had a cholestatic type, and 40 patients (12.7%) had a mixed type of liver injury, with the rest of 101 cases classified as unclear type due to lack of test results of alkaline phosphatase (ALP). The distribution of basic characteristics between ATLI cases and non-ATLI controls are summarized in Table 1 (The basic characteristics in Table 1 has been reported in our previous study)^21^. We used 1:2 individual matching of case: control, and there was no significant difference in age, sex and treatment history, disease severity and drug dosage between the two groups. Before anti-TB treatment, all patients’ liver biochemical parameters were in the normal range and there was no significant difference between the two groups (P > 0.05). However, during the treatment period, the peak serum alanine transaminase (ALT), aspartate aminotransaminase (AST) and total bilirubin levels were significantly higher in the ATLI group than in the controls (P < 0.001).

**Table 1:** Characteristics of patients in ATLI cases and non-ATLI controls.

Characteristic	ATLI cases	non-ATLI controls (n = 628)	*P* value
Sex (male/female)	238/76	476/152	—
Treatment history (primary/re-treatment)	283/31	566/62	—
Age (years)^a^	47.7 ± 19.0	47.6 ± 19.1	0.918^†^
Weight (Kg)^a^	56.3 ± 10.6	55.6 ± 10.0	0.003^†^
Hepatoprotectant (use/not use)	268/46	526/102	0.412^‡^
**Baseline value**			
ALT (U/L)^b^	16.0(15.0–24.0)	16.0(11.1–22.0)	0.090^¶^
AST (U/L)^b^	22.0(19.8–26.1)	22.0(17.0–27.0)	0.053^¶^
Total bilirubin (µmol/L)^b^	10.5(8.9–13.3)	10.5(7.7–13.3)	0.194^¶^
**During treatment (peak value)**			
ALT (U/L)^b^	120.0(89.0–191.5)	21.8(15.0–31.0)	<0.0001^¶^
AST (U/L)^b^	98.4(65.0–173.5)	27.0(21.0–34.1)	<0.0001^¶^
Total bilirubin (µmol/L)^b^	18.6(14.2–25.0)	13.0(9.8–17.5)	<0.0001^¶^

Abbreviations: ATLI, anti-tuberculosis drug-induced liver injury; ALT, alanine transaminase; AST, aspartate transaminase.

Normal range: ALT < 40 U/L, AST < 40 U/L, Total bilirubin <19 µmol/L.

^a^Values are presented as mean ± standard deviations.

^b^Values are presented as median (inter-quartile range).

^†^Two-factor analysis of variance test.

^‡^Conditional logistic regression model analysis.

^¶^Median test.

### Genotype analysis

No significant deviations from the Hardy-Weinberg equilibrium (HWE) in the distributions of genotypes and alleles were observed for the eleven tagSNPs among the control group [rs2886161, χ^2^ = 1.904, P = 0.168; rs4243387, χ^2^ = 0.479, P = 0.489; rs6726395, χ^2^ = 2.868, P = 0.090; rs1962142, χ^2^ = 0.002, P = 0.960; rs2001350, χ^2^ = 1.354, P = 0.245; rs1048290, χ^2^ = 2.064, P = 0.151; rs2267373, χ^2^ = 0.450, P = 0.502; rs4444637, χ^2^ = 1.456, P = 0.228; rs4821767, χ^2^ = 0.129, P = 0.720; rs4720833, χ^2^ = 0.289, P = 0.591 and rs3808337, χ^2^ = 0.362, P = 0.548], but not in the remaining two tagSNPs (rs11545829 and rs4608623) (Table 2).

**Table 2:** Information on thirteen tagSNPs of *NRF2*, *KEAP1*, *MAFF* and *MAFK*.

Gene	SNP NO.	Chromosome Position^†^	Location	Base Change	MAF^‡^	HWE *p*-value*
*NRF2*	rs2886161	178127839	intron1	C > T	44.4	0.168
	rs4243387	178117765	intron1	T > C	37.8	0.489
	rs6726395	178103229	intron1	G > A	43.0	0.090
	rs1962142	178113484	intron1	C > T	25.6	0.960
	rs2001350	178100425	intron1	A > G	32.6	0.245
*KEAP1*	rs1048290	10600442	exon4	G > C	44.4	0.151
	rs11545829	10599965	exon5	C > T	32.6	0.031
*MAFF*	rs2267373	38600542	intron1	T > C	35.7	0.502
	rs4608623	38597378	5′ near gene	G > T	47.0	<0.001
	rs4444637	38606780	intron1	G > A	10.5	0.228
	rs4821767	38614129	3′ near gene	A > C	43.0	0.720
*MAFK*	rs4720833	1574403	5′-UTR	G > A	36.9	0.591
	rs3808337	1576454	intron1	T > C	40.7	0.548

^†^SNP position in NCBI dbSNP (http://www.ncbi.nlm.nih.gov/projects/SNP).

^‡^Minor allele frequency (MAF) for Han Chinese in Beijing in the Hapmap database.

*Hardy-Weinberg equilibrium (HWE) *P-*value in the control group.

The genotype distributions of thirteen tagSNPs between ATLI cases and controls are shown in Table 3. Patients carrying TC genotype at rs4243387 in *NRF2* were at a higher risk of liver injury than with TT genotype (adjusted OR = 1.362, 95% CI: 1.017–1.824, P = 0.038). However, patients carrying TC genotype at rs2267373 in *MAFF* were at a lower risk of liver injury than with TT genotype (adjusted OR = 0.712, 95% CI: 0.532–0.953, P = 0.022), and these statistically significant differences were also found using a dominant model (P = 0.014) and an additive model (P = 0.022).

**Table 3:** Genotypes distribution in two groups and the risks of ATLI.

Gene	tagSNPs	ATLI Cases (N = 314)		non-ATLI controls (N = 628)		OR(95% CI)*	*P*	Model	OR(95% CI)*	*P*
		N	%	N	%					
*NRF2*	rs2886161(C > T)									
	CC	94	29.9	197	31.4	1.000		Dom	1.074(0.785–1.469)	0.654
	CT	155	49.4	294	46.8	1.114(0.799–1.553)	0.525	Rec	0.927(0.660–1.301)	0.661
	TT	65	20.7	137	21.8	0.992(0.665–1.480)	0.970	Add	1.003(0.823–1.222)	0.978
	rs4243387(T > C)									
	TT	148	47.1	336	53.5	1.000		Dom	1.299(0.980–1.723)	0.069
	TC	145	46.2	242	38.5	1.362(1.017–1.824)	0.038	Rec	0.831(0.483–1.432)	0.506
	CC	21	6.7	50	8.0	0.970(0.552–1.702)	0.915	Add	1.143(0.914–1.428)	0.241
	rs6726395(G > A)									
	GG	106	33.8	236	37.6	1.000		Dom	1.200(0.890–1.616)	0.231
	GA	163	51.9	314	50.0	1.174(0.862–1.599)	0.308	Rec	1.181(0.795–1.755)	0.410
	AA	45	14.3	78	12.4	1.311(0.841–2.043)	0.233	Add	1.151(0.931–1.424)	0.194
	rs1962142(C > T)									
	CC	160	51.0	349	55.6	1.000		Dom	1.213(0.911–1.616)	0.187
	CT	136	43.3	238	37.9	1.263(0.938–1.701)	0.124	Rec	0.857(0.481–1.524)	0.599
	TT	18	5.7	41	6.5	0.948(0.524–1.715)	0.861	Add	1.103(0.878–1.387)	0.399
	rs2001350(T > C)									
	TT	158	50.3	347	55.3	1.000		Dom	1.214(0.918–1.607)	0.174
	TC	134	42.7	232	36.9	1.259(0.940–1.686)	0.122	Rec	0.898(0.526–1.533)	0.693
	CC	22	7.0	49	7.8	0.995(0.574–1.726)	0.986	Add	1.108(0.889–1.380)	0.362
*KEAP1*	rs1048290(G > C)									
	GG	65	20.7	148	23.6	1.000		Dom	1.193(0.851–1.672)	0.306
	GC	176	56.1	332	52.8	1.227(0.861–1.749)	0.257	Rec	0.970(0.696–1.354)	0.860
	CC	73	23.2	148	23.6	1.119(0.738–1.695)	0.597	Add	1.056(0.860–1.297)	0.600
	rs11545829(C > T)									
	CC	139	44.3	273	43.5	1.000		Dom	0.966(0.725–1.286)	0.812
	CT	142	45.2	300	47.8	0.924(0.684–1.248)	0.606	Rec	1.234(0.779–1.954)	0.370
	TT	33	10.5	55	8.7	1.183(0.728–1.924)	0.498	Add	1.027(0.825–1.278)	0.813
*MAFF*	rs2267373(T > C)									
	TT	148	47.1	242	38.5	1.000		Dom	0.704(0.532–0.931)	0.014
	TC	131	41.7	302	48.1	0.712(0.532–0.953)	0.022	Rec	0.813(0.530–1.249)	0.345
	CC	35	11.2	84	13.4	0.671(0.423–1.062)	0.088	Add	0.782(0.633–0.965)	0.022
	rs4608623(G > T)									
	GG	98	31.2	232	36.9	1.000		Dom	1.337(0.979–1.825)	0.068
	GT	137	43.6	260	41.4	1.290(0.922–1.804)	0.137	Rec	1.225(0.885–1.695)	0.220
	TT	79	25.2	136	21.7	1.426(0.971–2.094)	0.070	Add	1.197(0.989–1.449)	0.064
	rs4444637(G > A)									
	GG	256	81.5	491	78.2	1.000		Dom	0.824(0.587–1.155)	0.260
	GA	51	16.2	125	19.9	0.794(0.557–1.132)	0.203	Rec	1.188(0.454–3.104)	0.726
	AA	7	2.3	12	1.9	1.143(0.437–2.992)	0.785	Add	0.875(0.651–1.176)	0.377
	rs4821767(A > C)									
	AA	84	26.8	134	21.3	1.000		Dom	0.753(0.553–1.026)	0.073
	AC	155	49.4	317	50.5	0.789(0.569–1.093)	0.155	Rec	0.803(0.586–1.098)	0.169
	CC	75	23.9	177	28.2	0.682(0.464–1.002)	0.051	Add	0.826(0.681–1.001)	0.051
*MAFK*	rs4720833(G > A)									
	GG	148	47.1	316	50.3	1.000		Dom	1.142(0.866–1.504)	0.347
	GA	139	44.3	255	40.6	1.168(0.877–1.557)	0.289	Rec	0.935(0.571–1.529)	0.788
	AA	27	8.6	57	9.1	1.010(0.604–1.687)	0.971	Add	1.069(0.863–1.325)	0.539
	rs3808337(T > C)									
	TT	144	45.9	297	47.3	1.000		Dom	1.060(0.804–1.399)	0.678
	TC	141	44.9	265	42.2	1.095(0.821–1.461)	0.536	Rec	0.863(0.543–1.373)	0.535
	CC	29	9.2	66	10.5	0.906(0.555–1.477)	0.692	Add	1.003(0.812–1.238)	0.978

Abbreviations: ATLI, anti-tuberculosis drug-induced liver injury; Dom, dominant model; Rec, recessive model; Add, additive model.

*Conditional logistic regression model analysis and adjusted for weight and usage of hepatoprotectant.

### Haplotype analysis

**Figure 1:**
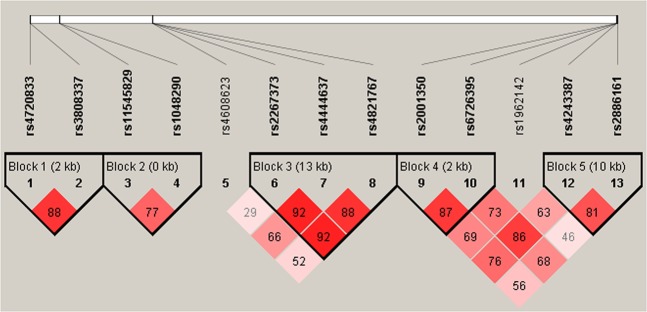
Linkage disequilibrium (LD) block constructed from 13 tagSNPs in *NRF2*, *KEAP1*, *MAFF* and *MAFK*. This LD plot was generated with the Haploview 4.2 software. Markers with LD (D′ < 1 and LOD > 2) are shown in red through pink (color intensity decreases with decreasing D′ value). D′ value shown within each square represents a pairwise LD relationship between the two polymorphisms.

Five potential linkage disequilibrium (LD) blocks were constructed based on the r-square value and log-odds score (Fig. 1), and statistical analysis results indicated that patients carrying haplotype C-C in block 4 (rs2001350–rs6726395, in *NRF2*) had a higher risk of liver injury (adjusted OR = 2.503, 95% CI: 1.273–4.921, P = 0.008), and patients carrying haplotype C-G-C in block 3 (rs2267373-rs4444637-rs4821767, in *MAFF*) had a lower risk of liver injury (adjusted OR = 0.753, 95% CI: 0.587–0.965, P = 0.025) (Table 4).

**Table 4:** Haplotype frequencies in two groups and the risks of ATLI.

Gene	Haplotypes	ATLI cases (%)	Non-ATLI controls (%)	OR(95% CI)*	*P*
*NRF2*	rs4243387-rs2886161				
	T-G	57.80	60.83	1	
	C-A	26.43	24.52	1.133(0.901–1.425)	0.286
	T-A	13.85	12.90	1.136(0.851–1.515)	0.387
	C-G	1.91	1.75	1.116(0.538–2.317)	0.768
	rs2001350-rs6726395				
	T-C	51.11	53.10		
	C-T	26.27	25.56	1.051(0.831–1.329)	0.679
	T-T	19.11	19.67	0.990(0.766–1.278)	0.937
	C-C	3.50	1.67	2.503(1.273–4.921)	0.008
*KEAP1*	rs11545829-rs1048290				
	C-G	46.02	47.29	1	
	T-C	30.41	29.94	1.042(0.833–1.304)	0.718
	C-C	20.86	20.06	1.064(0.826–1.370)	0.633
	C-G	2.71	2.71	1.041(0.540–2.006)	0.905
*MAFF*	rs2267373-rs4444637-rs4821767				
	T-G-A	51.16	45.72	1	
	C-G-C	20.99	25.38	0.753(0.587–0.965)	0.025
	T-G-C	17.95	16.73	0.968(0.742–1.265)	0.814
	C-A-C	9.78	11.37	0.797(0.572–1.109)	0.177
	C-G-A	1.12	0.80	1.320(0.499–3.491)	0.575
*MAFK*	rs4720833-rs3808337				
	G-T	66.24	65.61	1	
	A-C	27.23	28.03	1.039 (0.834–1.295)	0.733
	G-C	4.38	3.66	0.842(0.511–1.389)	0.501
	A-T	2.15	2.71	1.274(0.677–2.397)	0.453

Abbreviations: ATLI, anti-tuberculosis drug-induced liver injury.

*Conditional logistic regression model and adjusted for weight and usage of hepatoprotectant.

### Subgroup analysis

The association between tagSNPs and ATLI among different clinical patterns of liver injury are shown in Supplementary Tables 1, 2, 3 and 4. Patients carrying polymorphisms of rs4444637 in *MAFF* had a reduced risk of hepatocellular liver injury (dominant model, adjusted OR = 0.601, 95% CI: 0.363–0.994, P = 0.047), and a similar relationship existed between polymorphisms of rs4821767 in *MAFF* and cholestatic liver injury (dominant model, adjusted OR = 0.250, 95% CI: 0.074–0.841, P = 0.025; additive model, adjusted OR = 0.353, 95% CI: 0.142–0.878, P = 0.025). However, patients carrying polymorphisms of rs4720833 in *MAFK* had an increased risk of mixed liver injury (recessive model, adjusted OR = 4.127, 95% CI: 1.054–16.16, P = 0.042; additive model, adjusted OR = 2.000, 95% CI: 1.096–3.650, P = 0.024) (Table 5).

**Table 5:** Genotypes distribution in two groups among different clinical pattern of liver injury.

Gene	tag SNPs	Model	Hepatocellular(n = 150)		Cholestatic(n = 23)		Mixed(n = 40)		Unclear(n = 101)	
			OR(95% CI)*	*P*	OR(95% CI)*	*P*	OR(95% CI)*	*P*	OR(95% CI)*	*P*
*NRF2*	rs2886161(C > T)									
	CC	Dom	1.217(0.763–1.943)	0.410	1.122(0.338–3.722)	0.851	1.387(0.573–3.355)	0.468	0.818(0.475–1.408)	0.468
	CT	Rec	0.863(0.535–1.391)	0.545	0.613(0.138–2.722)	0.520	0.632(0.222–1.801)	0.390	1.302(0.716–2.367)	0.388
	TT	Add	1.021(0.770–1.352)	0.887	0.903(0.400–2.041)	0.807	0.998(0.565–1.764)	0.995	1.006(0.710–1.426)	0.973
	rs4243387(T > C)									
	TT	Dom	1.019(0.679–1.530)	0.928	0.651(0.246–1.718)	0.385	1.914(0.823–4.451)	0.132	2.146(1.246–3.697)	0.006
	TC	Rec	0.968(0.426–2.199)	0.937	—	0.989	0.952(0.244–3.710)	0.943	0.855(0.332–2.203)	0.746
	CC	Add	1.007(0.725–1.398)	0.967	0.585(0.250–1.369)	0.217	1.436(0.768–2.686)	0.257	1.530(1.018–2.301)	0.410
	rs6726395(G > A)									
	GG	Dom	1.145(0.740–1.771)	0.543	0.777(0.293–2.064)	0.613	1.469(0.604–3.572)	0.397	1.422(0.828–2.443)	0.202
	GA	Rec	1.213(0.707–2.083)	0.483	0.638(0.123–3.320)	0.594	1.238(0.323–4.748)	0.755	1.262(0.602–2.646)	0.539
	AA	Add	1.134(0.838–1.535)	0.416	0.797(0.387–1.644)	0.540	1.347(0.670–2.705)	0.403	1.274(0.869–1.868)	0.215
	rs1962142(C > T)									
	CC	Dom	1.069(0.710–1.608)	0.750	1.269(0.479–3.362)	0.632	1.445(0.631–3.306)	0.384	1.454(0.852–2.483)	0.170
	CT	Rec	1.154(0.510–2.612)	0.731	—	0.989	0.694(0.123–3.907)	0.679	0.817(0.304–2.198)	0.689
	TT	Add	1.070(0.767–1.491)	0.691	0.952(0.427–2.121)	0.905	1.196(0.619–2.311)	0.594	1.201(0.801–1.800)	0.376
	rs2001350(T > C)									
	TT	Dom	0.980(0.652–1.473)	0.923	0.643(0.229–1.808)	0.402	2.361(0.946–5.896)	0.066	1.560(0.962–2.530)	0.072
	TC	Rec	1.017(0.454–2.279)	0.967	—	0.982	1.200(0.309–4.669)	0.792	0.929(0.371–2.324)	0.874
	CC	Add	0.990(0.712–1.376)	0.951	0.570(0.243–1.336)	0.196	1.712(0.866–3.384)	0.122	1.291(0.891–1.871)	0.177
*KEAP1*	rs1048290(G > C)									
	GG	Dom	1.439(0.872–2.374)	0.155	0.816(0.232–2.875)	0.752	0.716(0.251–2.041)	0.532	1.297(0.720–2.336)	0.386
	GC	Rec	1.350(0.852–2.137)	0.201	0.368(0.094–1.439)	0.151	0.585(0.164–2.088)	0.409	0.847(0.460–1.558)	0.593
	CC	Add	1.280(0.954–1.718)	0.100	0.633(0.286–1.397)	0.257	0.701(0.333–1.477)	0.350	1.043(0.725–1.499)	0.821
	rs11545829(C > T)									
	CC	Dom	1.067(0.706–1.613)	0.759	0.778(0.284–2.136)	0.627	0.830(0.362–1.902)	0.659	0.928(0.547–1.574)	0.782
	CT	Rec	1.630(0.895–2.970)	0.110	—	0.991	2.744(0.454–16.57)	0.271	0.796(0.335–1.892)	0.605
	TT	Add	1.167(0.864–1.576)	0.315	0.652(0.260–1.633)	0.361	1.023(0.496–2.108)	0.951	0.913(0.615–1.356)	0.653
*MAFF*	rs2267373(T > C)									
	TT	Dom	0.719(0.480–1.077)	0.110	0.434(0.137–1.378)	0.157	0.938(0.428–2.056)	0.873	0.639(0.387–1.056)	0.080
	TC	Rec	0.656(0.345–1.249)	0.200	0.786(0.137–4.516)	0.787	0.486(0.098–2.404)	0.376	1.137(0.567–2.279)	0.717
	CC	Add	0.758(0.560–1.026)	0.073	0.560(0.226–1.387)	0.210	0.838(0.441–1.592)	0.589	0.818(0.568–1.178)	0.280
	rs4608623(G > T)									
	GG	Dom	1.154(0.730–1.825)	0.539	1.565(0.428–5.716)	0.498	0.947(0.384–2.334)	0.906	1.837(1.075–3.139)	0.026
	GT	Rec	1.005(0.640–1.579)	0.982	0.983(0.314–3.076)	0.976	1.351(0.438–4.167)	0.601	1.923(1.027–3.601)	0.041
	TT	Add	1.057(0.801–1.395)	0.695	1.134(0.570–2.254)	0.720	1.073(0.560–2.054)	0.832	1.525(1.089–2.135)	0.014
	rs4444637(G > A)									
	GG	Dom	0.601(0.363–0.994)	0.047	0.309(0.072–1.320)	0.113	1.416(0.528–3.793)	0.489	1.261(0.703–2.262)	0.436
	GA	Rec	0.787(0.194–3.188)	0.738	—	0.989	1.900(0.107–33.81)	0.662	5.742(0.593–55.56)	0.131
	AA	Add	0.664(0.428–1.032)	0.069	0.321(0.078–1.320)	0.115	1.384(0.580–3.300)	0.464	1.365(0.804–2.317)	0.249
	rs4821767(A > C)									
	AA	Dom	0.863(0.550–1.354)	0.522	0.250(0.074–0.841)	0.025	0.986(0.356–2.734)	0.978	0.709(0.416–1.208)	0.206
	AC	Rec	0.757(0.476–1.205)	0.241	0.397(0.081–1.960)	0.257	0.898(0.351-2.301)	0.823	0.890(0.530–1.495)	0.660
	CC	Add	0.848(0.638–1.127)	0.256	0.353(0.142–0.878)	0.025	0.950(0.513–1.758)	0.870	0.849(0.618–1.168)	0.315
*MAFK*	rs4720833(G > A)									
	GG	Dom	0.875(0.582–1.314)	0.519	1.165(0.416–3.262)	0.771	2.037(0.937–4.429)	0.073	1.347(0.825–2.199)	0.234
	GA	Rec	0.820(0.390–1.720)	0.599	—	0.980	4.127(1.054–16.16)	0.042	0.797(0.317–2.002)	0.629
	AA	Add	0.885(0.640–1.222)	0.458	0.814(0.367–1.807)	0.613	2.000(1.096–3.650)	0.024	1.150(0.788–1.677)	0.469
	rs3808337(T > C)									
	TT	Dom	0.754(0.503–1.130)	0.171	1.620(0.534–4.911)	0.394	1.476(0.670–3.248)	0.334	1.398(0.855–2.284)	0.181
	TC	Rec	0.606(0.285–1.286)	0.192	—	0.978	2.816(0.783–10.12)	0.113	1.096(0.515–2.333)	0.812
	CC	Add	0.763(0.555–1.051)	0.098	0.925(0.427–2.004)	0.844	1.588(0.864–2.918)	0.136	1.225(0.855–1.757)	0.269

Abbreviations: ATLI, anti-tuberculosis drug-induced liver injury; Dom, dominant model; Rec, recessive model; Add, additive model.

*Conditional logistic regression model and adjusted for weight and usage of hepatoprotectant.

## Discussion

In present study, the role of 13 tagSNPs in *NRF2*, *KEAP1*, *MAFF* and *MAFK* in the Nrf2-ARE signaling pathway were examined among Chinese anti-TB treatment patients. Two single variants, namely, the TC genotype at rs4243387 in *NRF2* and TC genotype at rs2267373 in *MAFF*, together with two haplotypes of C-C (rs2001350-rs6726395, in *NRF2*) and C-G-C (rs2267373-rs4444637-rs4821767, in *MAFF*), were identified as being associated with ATLI development. To our knowledge, there has been only one study conducted in Japanese to explore the relationship of the genetic polymorphisms in the oxidative stress signaling pathway and the occurrence of ATLI^22^. In that study, Nanashima *et al*. performed a candidate gene-based association study between thirty-four tagSNPs in 10 genes in the antioxidant pathway and ATLI susceptibility with 18 ATLI patients and 82 controls^22^. The results revealed that a CC genotype at rs11080344 in nitric oxide synthase 2A (*NOS2A*), a CC genotype at rs2070401 in BTB domain and CNC homologue 1 (*BACH1*), and a GA or AA genotype at rs4720833 in *MAFK* independently conferred ATLI susceptibility^22^. Together with the Japanese study, the present study further confirms the role of related genetic polymorphisms of the Nrf2-ARE signaling pathway in ATLI.

Nrf2 is a central regulator which mediates antioxidant gene expression^23^, and a potential target to prevent or cure drug-induced liver injury^24^. Our study suggested that a TC genotype at rs4243387 in *NRF2* is associated with increased risk of ATLI (Adjusted OR = 1.362, 95% CI: 1.017–1.824, P = 0.038). A previous functional study of polymorphisms in *NRF2* indicated that genetic variants would lead to a significant reduction of *NRF2* gene expression and a less efficient binding of Nrf2 to ARE, and increase the risk of acute lung injury^25^. However, the same variant was not found to be statistically significant in the Japanese study, which may be related to the low sample size^22^. The sMafs are crucial regulators of mammalian gene expression that are essential for DNA binding of Nrf2 and other processes, including the localization and stabilization of Nrf2^19,26^. Our study indicated that TC genotype at rs2267373 in *MAFF* was associated with reduced risk of ATLI (Adjusted OR = 0.712, 95% CI: 0.532–0.953, P = 0.022); significant differences were also found using a dominant model (P = 0.014) and an additive model (P = 0.022). Other two tagSNPs (rs4821767 and rs4444637) in *MAFF* were also associated with reduced risk of specific clinical types of liver injury under different genetic models. However, the function of tagSNPs rs2267373, rs4821767 and rs4444637 in intron 1 of *MAFF* remains unknown. We performed bioinformatics analysis of three tagSNPs in *MAFF* using online database (HaploReg v4.1)^27^, and the result indicated that rs2267373, rs4821767 and rs4444637 contained H3K4me1 and H3K27ac in liver tissue, and appear to change known motifs. H3K4me1 and H3K27ac are the predominant histone modification found in nucleosomes around enhancer element, and associated with transcriptional regulation of genes^28^. Perhaps the variants in *MAFF* could regulate the expression of *MAFF*. Higher expression of *MAFF* facilitates it binding to Nrf2, leading to increased expression of subsequent antioxidant enzymes and reducing the occurrence of ATLI^26^. The role of these genetic variations in ATLI needs further research.

Although both the present study and Japanese study revealed that genetic polymorphisms in Nrf2-ARE signaling pathway may contribute to the susceptibility to ATLI, there are some difference between two studies. Our study indicated that tagSNP rs2267373 in *MAFF* was associated with a reduced susceptibility to ATLI (Dominant model, Adjusted OR = 0.704, 95% CI: 0.532–0.931, P = 0.014), whereas the Japanese study conferred tagSNP rs4720833 in *MAFK* was associated with an increased susceptibility to ATLI (Dominant model, OR = 3.162, 95% CI: 1.033–9.686, P = 0.037)^22^. In the present study, rs4720833 in *MAFK* was found to increase the risk of ATLI only in mixed patients under a recessive model (adjusted OR = 4.127, 95% CI: 1.054–16.16, P = 0.042) and an additive model (adjusted OR = 2.000, 95% CI: 1.096–3.650, P = 0.024), but not under a dominant model. Because of sequence similarity, no functional differences have been observed among the sMafs (MafF, MafG and MafK) in terms of their bZIP structures^29^. The sequencing results of each *MAF* gene suggested that *MAFF* was the most polymorphic of the *MAF* genes followed by *MAFK*, whereas *MAFG* had the lowest molecular plasticity^30^. Additionally, animal studies have also revealed that maff and mafk knockout mice, as well as the double knockout maff:mafk, did not have major phenotypical effects^31^. The accumulating lines of evidence unequivocally illustrated the importance and complexity of sMafs in the CNC-sMaf transcription factor network. Further research is needed on the role of these genetic variants in liver injury, especially in different ethnic populations.

This study was a nested case-control design based on anti-TB treatment cohort that decreases recall bias. The ATLI sample size in the present study was relatively large (more than our estimated minimum sample size), which allowed us to increase efficiency and control potential confounders by performing 1:2 matching. Moreover, each case was strictly assessed by experts to minimize the misclassification of diagnosis. However, there were several limitations in our study. First, we did not collect the patient histories of previous hepatitis C infection, which may affect the occurrence of liver injury. Second, due to the combination therapy strategy, we cannot explain the pathogenic mechanisms of specific drugs.

In summary, it is important to determine the relationship between genetic polymorphisms of *NRF2*, *KEAP1*, *MAFF*, *MAFK* and the risk of ATLI in the Chinese population and which genetic polymorphisms of *NRF2*, *MAFF* and *MAFK* may contribute to the susceptibility to ATLI. Furthermore, new studies in larger and varied populations are required to validate these relationships.

## Methods

### Anti-TB patients’ recruitment and follow-up

The study patients were recruited from the outpatient departments of four designated TB diagnosis and treatment hospitals between April 2014 and December 2016 based on the ADACS protocol^32^. This cohort of anti-TB treatment patients has been described in our previous study^21^. In brief, before treatment, patients would finish the baseline questionnaire (sex, age, TB treatment history, sputum smear, and other complications) and receive laboratory examinations, including serum hepatitis B virus surface antigen (HBsAg), ALT, AST, direct and total bilirubin levels. TB patients received the standard anti-TB short course chemotherapy regimen under DOTS strategy, including RIF, INH, PZA, EMB and/or streptomycin (SM/S), specifically 2HRZE/4HR for primary patients and 2HRZES/6HRE for retreatment patients^32^. Patients were monitored for 6~9 months according to the treatment episode. During the anti-TB treatment, a method combining active self-recorded diaries and passive scheduled liver function tests was used to detect abnormal liver function in time. Patients were asked to self-record signs and/or symptoms of discomfort and the local supervising doctors often checked the records for potential ATLI. Patients also received the scheduled liver function tests every two weeks in the first two months of treatment or when patients had exhibited some symptoms of suspected hepatic toxicity^32^.

Patients with one or more of the following were excluded: (i) patients with abnormal serum ALT, AST or total bilirubin levels before treatment; (ii) patients with HBsAg (+) serum; (iii) patients with alcoholic liver disease; (iv) patients with concomitant use of hepatotoxic drugs or habitual alcohol consumption; and (v) patients with chronic liver disease or other diseases that can also cause elevated liver enzymes.

The study was approved by the Ethics Committee of Nanjing Medical University and conducted in accordance with the Declaration of Helsinki Principles. Written informed consent was obtained from all patients.

### ATLI Cases and non-ATLI controls selection and matching

A nested case-control study was conducted based on the dynamic cohort. The diagnostic criteria of ATLI was proposed by the international consensus meeting, namely, an increase in ALT levels greater than two-times of the upper limit of normal (ULN), with/without a combined increase in AST and total bilirubin levels, provided that one of them was more than two-times of ULN during the treatment^33^. Furthermore, the causality assessment result was certain, probable or possible based on the WHO Uppsala Monitoring Center system^34^. Each ATLI case was also strictly reviewed by experts from the local ADR monitoring center. Pattern of ATLI was defined by R value where R = (ALT/ULN)/(ALP/ULN)^35^. If R ≥ 5, then the pattern was hepatocellular. If R > 2 and <5, then the pattern was mixed. If R ≤ 2, then the pattern was cholestatic.

The patients who did not meet the ATLI criteria were considered candidate controls. For every ALTI case, two control patients were matched by sex, age (±5 years), treatment history, disease severity and drug dosage.

### Sample size calculation

The sample size in present study was calculated using the Quanto statistical program (version 1.2.4, University of Southern California, USA)^36^. Based on our previous matched case-control study of ATLI, the effect size (odds ratio) was set at 2.0 with at least 90 percent power under the dominance model. Moreover, the minor allele frequency (MAF) was set at 10 percent, with a type I error level of 0.05. The incidence of ATLI in the Chinese anti-TB treatment population was 11.9%^32^. Finally, the sample size of the two groups was 253 ATLI cases and 253 non-ATLI controls.

### TagSNPs selection and genotyping

TagSNPs in five genes (*NRF2*, *KEAP1*, *MAFF*, *MAFK* and *MAFG*) were selected from the Haploview software 4.2 (Broad Institute, Cambridge, MA, USA), based on the Chinese Han population data of Hapmap and the following criteria: (i) MAF ≥5% in Chinese population; (ii) r-square of pairwise linkage disequilibrium (LD) ≥0.8. As a result, fourteen potential tagSNPs were selected for genotyping using the Sequenom MassARRAY iplex Platform (Sequenom Inc., Hamburg, Germany). However, one tagSNP in *MAFG* (rs148165792, MAF = 7.3%) was excluded from the study due to a failed probe design. Technicians who performed the genotyping were blinded to the status of case and control. More than 10% of samples were selected randomly for repeated experiments with repeatability of 100%. The overall genotyping success rate was 100%. As a result, 13 tagSNPs of four genes were analyzed in present study (Table 2).

### Statistical analysis

Distributions of demographic and clinical characteristics among two groups were evaluated by two-factor analysis of variance test (for normal continuous variables) or nonparametric test (for non-normal continuous variables), or by chi-square test (for categorical variables). Hardy-Weinberg equilibrium (HWE) in the control group was assessed by the chi-square test. Haploview software 4.2 was used to select haplotype blocks in consideration of the LD between SNPs in each gene. PHASE 2.1 was used to estimate haplotype frequencies for different gene. Multivariate conditional logistic regression model was used to analysis the genotype frequency differences between two groups. Three different genetic models (additive model, dominant model and recessive model) were used to comprehensively analyze the effect of tagSNPs. The association between genotypes and the risk of ATLI was estimated by odds ratios (ORs) and 95% confidence intervals (CIs), with weight and usage of hepatoprotectant as covariates. All statistical analyses were performed using the SPSS 20.0 (IBM Inc., Chicago, IL, USA). A two-tailed P-value < 0.05 was considered statistically significant.

## Supplementary information

Supplementary File
