# Supplementary material for: Association between genetic polymorphisms of NRF2, KEAP1, MAFF, MAFK and anti-tuberculosis drug-induced liver injury: a nested case-control study
Source: Sci Rep. 2019 Oct 4;9:14311. doi: 10.1038/s41598-019-50706-y (PMC6778130; doi:10.1038/s41598-019-50706-y)
Supplement: Supplementary file 1 — Supplementary File [file 41598_2019_50706_MOESM1_ESM.docx]

**Association between genetic polymorphisms of NRF2, KEAP1, MAFF, MAFK and anti-tuberculosis drug-induced liver injury: a nested case-control study**

Shixian Chen^1^, Hongqiu Pan^2^, Yongzhong Chen^2^, Lihuan Lu^3^, Xiaomin He^4^, Hongbo Chen^5^, Ru Chen^6^, Siyan Zhan^6^ & Shaowen Tang^1^

1. Department of Epidemiology, School of Public Health, Nanjing Medical University, Nanjing, 211166, China
2. Department of tuberculosis, The third people’s hospital of Zhenjiang affiliated to Jiangsu University, Zhenjiang 212005, China
3. Department of tuberculosis, The second people’s hospital of Changshu; Changshu 215500, China
4. Department of infectious Disease, The people’s hospital of Taixing, Taixing 225400, China
5. Department of infectious Disease, The people’s hospital of Jurong, Jurong 212400, China
6. Department of Epidemiology and Biostatistics, School of Public Health, Peking University Health Science Centre, Beijing, 100191, China

**Corresponding Author:**

Associate Professor Shaowen Tang.

Department of Epidemiology, School of Public Health, Nanjing Medical University, Nanjing, 211166, China

818 Tianyuandong Road, Jiangning District, Nanjing, P.R.China, 211166.

Office: 86-25-86868224. Fax: 86-25-86868499.

E-mail: tomswen@njmu.edu.cn.

**Supplementary Table 1** Genotypes distribution in ATLI cases and non-ATLI controls in **hepatocellular** liver injury cases

| Gene | tagSNPs | ATLI Cases (N=150) | |  | non-ATLI controls (N=300) | | OR(95%CI)^*^ | *P* | Model | OR(95%CI)^*^ | *P* |
| --- | --- | --- | --- | --- | --- | --- | --- | --- | --- | --- | --- |
|  |  | N | % |  | N | % |  |  |  |  |  |
| *NRF2* | rs2886161(C>T) |  |  |  |  |  |  |  |  |  |  |
|  | CC | 38 | 25.3 |  | 86 | 28.7 | 1.000 |  | Dom | 1.217(0.763-1.943) | 0.410 |
|  | CT | 81 | 54.0 |  | 145 | 48.3 | 1.334(0.807-2.205) | 0.261 | Rec | 0.863(0.535-1.391) | 0.545 |
|  | TT | 31 | 20.7 |  | 69 | 23.0 | 1.033(0.581-1.836) | 0.911 | Add | 1.021(0.770-1.352) | 0.887 |
|  | rs4243387(T>C) |  |  |  |  |  |  |  |  |  |  |
|  | TT | 79 | 52.0 |  | 156 | 52.0 | 1.000 |  | Dom | 1.019(0.679-1.530) | 0.928 |
|  | TC | 62 | 41.3 |  | 124 | 41.3 | 1.026(0.671-1.568) | 0.906 | Rec | 0.968(0.426-2.199) | 0.937 |
|  | CC | 10 | 6.7 |  | 20 | 6.7 | 0.978(0.422-2.265) | 0.958 | Add | 1.007(0.725-1.398) | 0.967 |
|  | rs6726395(G>A) |  |  |  |  |  |  |  |  |  |  |
|  | GG | 49 | 32.7 |  | 106 | 35.3 | 1.000 |  | Dom | 1.145(0.740-1.771) | 0.543 |
|  | GA | 75 | 50.0 |  | 151 | 50.3 | 1.107(0.704-1.740) | 0.660 | Rec | 1.213(0.707-2.083) | 0.483 |
|  | AA | 26 | 17.3 |  | 43 | 14.4 | 1.298(0.699-2.410) | 0.409 | Add | 1.134(0.838-1.535) | 0.416 |
|  | rs1962142(C>T) |  |  |  |  |  |  |  |  |  |  |
|  | CC | 86 | 57.3 |  | 177 | 59.0 | 1.000 |  | Dom | 1.069(0.710-1.608) | 0.750 |
|  | CT | 54 | 36.0 |  | 106 | 35.3 | 1.052(0.688-1.609) | 0.814 | Rec | 1.154(0.510-2.612) | 0.731 |
|  | TT | 10 | 6.7 |  | 17 | 5.7 | 1.181(0.510-2.737) | 0.697 | Add | 1.070(0.767-1.491) | 0.691 |
|  | rs2001350(T>C) |  |  |  |  |  |  |  |  |  |  |
|  | TT | 80 | 53.3 |  | 159 | 53.0 | 1.000 |  | Dom | 0.980(0.652-1.473) | 0.923 |
|  | TC | 59 | 39.3 |  | 119 | 39.7 | 0.976(0.639-1.491) | 0.911 | Rec | 1.017(0.454-2.279) | 0.967 |
|  | CC | 11 | 7.4 |  | 22 | 7.3 | 1.006(0.439-2.307) | 0.989 | Add | 0.990(0.712-1.376) | 0.951 |
| *KEAP1* | rs1048290(G>C) |  |  |  |  |  |  |  |  |  |  |
|  | GG | 30 | 20.0 |  | 77 | 25.7 | 1.000 |  | Dom | 1.439(0.872-2.374) | 0.155 |
|  | GC | 75 | 50.0 |  | 150 | 50.0 | 1.336(0.786-2.272) | 0.285 | Rec | 1.350(0.852-2.137) | 0.201 |
|  | CC | 45 | 30.0 |  | 73 | 24.3 | 1.647(0.910-2.979) | 0.099 | Add | 1.280(0.954-1.718) | 0.100 |
|  | rs11545829(C>T) |  |  |  |  |  |  |  |  |  |  |
|  | CC | 68 | 45.3 |  | 140 | 46.7 | 1.000 |  | Dom | 1.067(0.706-1.613) | 0.759 |
|  | CT | 60 | 40.0 |  | 130 | 43.3 | 0.950(0.610-1.477) | 0.818 | Rec | 1.630(0.895-2.970) | 0.110 |
|  | TT | 22 | 14.7 |  | 30 | 10.0 | 1.588(0.837-3.012) | 0.157 | Add | 1.167(0.864-1.576) | 0.315 |
| *MAFF* | rs2267373(T>C) |  |  |  |  |  |  |  |  |  |  |
|  | TT | 70 | 46.7 |  | 116 | 38.7 | 1.000 |  | Dom | 0.719(0.480-1.077) | 0.110 |
|  | TC | 64 | 42.7 |  | 138 | 46.0 | 0.763(0.499-1.167) | 0.212 | Rec | 0.656(0.345-1.249) | 0.200 |
|  | CC | 16 | 10.6 |  | 46 | 15.3 | 0.571(0.289-1.125) | 0.105 | Add | 0.758(0.560-1.026) | 0.073 |
|  | rs4608623(G>T) |  |  |  |  |  |  |  |  |  |  |
|  | GG | 36 | 24.0 |  | 81 | 27.0 | 1.000 |  | Dom | 1.154(0.730-1.825) | 0.539 |
|  | GT | 76 | 50.7 |  | 144 | 48.0 | 1.171(0.723-1.898) | 0.521 | Rec | 1.005(0.640-1.579) | 0.982 |
|  | TT | 38 | 25.3 |  | 75 | 25.0 | 1.120(0.639-1.962) | 0.693 | Add | 1.057(0.801-1.395) | 0.695 |
|  | rs4444637(G>A) |  |  |  |  |  |  |  |  |  |  |
|  | GG | 125 | 83.3 |  | 224 | 74.7 | 1.000 |  | Dom | 0.601(0.363-0.994) | 0.047 |
|  | GA | 22 | 14.7 |  | 68 | 22.7 | 0.586(0.344-0.998) | 0.049 | Rec | 0.787(0.194-3.188) | 0.738 |
|  | AA | 3 | 2.0 |  | 8 | 2.6 | 0.737(0.182-2.983) | 0.669 | Add | 0.664(0.428-1.032) | 0.069 |
|  | rs4821767(A>C) |  |  |  |  |  |  |  |  |  |  |
|  | AA | 38 | 25.3 |  | 67 | 22.3 | 1.000 |  | Dom | 0.863(0.550-1.354) | 0.522 |
|  | AC | 76 | 50.7 |  | 144 | 48.0 | 0.926(0.579-1.479) | 0.747 | Rec | 0.757(0.476-1.205) | 0.241 |
|  | CC | 36 | 24.0 |  | 89 | 29.7 | 0.716(0.403-1.272) | 0.255 | Add | 0.848(0.638-1.127) | 0.256 |
| *MAFK* | rs4720833(G>A) |  |  |  |  |  |  |  |  |  |  |
|  | GG | 76 | 50.7 |  | 143 | 47.7 | 1.000 |  | Dom | 0.875(0.582-1.314) | 0.519 |
|  | GA | 63 | 42.0 |  | 130 | 43.3 | 0.893(0.586-1.361) | 0.599 | Rec | 0.820(0.390-1.720) | 0.599 |
|  | AA | 11 | 7.3 |  | 27 | 9.0 | 0.771(0.355-1.674) | 0.511 | Add | 0.885(0.640-1.222) | 0.458 |
|  | rs3808337(T>C) |  |  |  |  |  |  |  |  |  |  |
|  | TT | 74 | 49.4 |  | 129 | 43.0 | 1.000 |  | Dom | 0.754(0.503-1.130) | 0.171 |
|  | TC | 65 | 43.3 |  | 138 | 46.0 | 0.801(0.527-1.217) | 0.298 | Rec | 0.606(0.285-1.286) | 0.192 |
|  | CC | 11 | 7.3 |  | 33 | 11.0 | 0.540(0.248-1.179) | 0.122 | Add | 0.763(0.555-1.051) | 0.098 |

Abbreviations: ATLI, anti-tuberculosis drug-induced liver injury. Dom, dominant model; Rec, recessive model; Add, additive model.

^*^Conditional logistic regression model analysis and adjusted for weight and usage of hepatoprotectant.

**Supplementary Table 2** Genotypes distribution in ATLI cases and non-ATLI controls in **Cholestatic** liver injury cases

| Gene | tagSNPs | ATLI Cases (N=23) | |  | non-ATLI controls (N=46) | | OR(95%CI)^*^ | *P* | Model | OR(95%CI)^*^ | *P* |
| --- | --- | --- | --- | --- | --- | --- | --- | --- | --- | --- | --- |
|  |  | N | % |  | N | % |  |  |  |  |  |
| *NRF2* | rs2886161(C>T) |  |  |  |  |  |  |  |  |  |  |
|  | CC | 5 | 21.7 |  | 11 | 23.9 | 1.000 |  | Dom | 1.122(0.338-3.722) | 0.851 |
|  | CT | 15 | 65.2 |  | 26 | 56.5 | 1.247(0.366-4.240) | 0.724 | Rec | 0.613(0.138-2.722) | 0.520 |
|  | TT | 3 | 13.1 |  | 9 | 19.6 | 0.712(0.126-4.015) | 0.701 | Add | 0.903(0.400-2.041) | 0.807 |
|  | rs4243387(T>C) |  |  |  |  |  |  |  |  |  |  |
|  | TT | 13 | 56.5 |  | 20 | 43.5 | 1.000 |  | Dom | 0.651(0.246-1.718) | 0.385 |
|  | TC | 10 | 43.5 |  | 22 | 47.8 | 0.843(0.292-2.436) | 0.752 | Rec | - | 0.989 |
|  | CC | 0 | 0.0 |  | 4 | 8.7 | - | 0.984 | Add | 0.585(0.250-1.369) | 0.217 |
|  | rs6726395(G>A) |  |  |  |  |  |  |  |  |  |  |
|  | GG | 10 | 43.5 |  | 16 | 34.8 | 1.000 |  | Dom | 0.777(0.293-2.064) | 0.613 |
|  | GA | 11 | 47.8 |  | 24 | 52.2 | 0.843(0.292-2.430) | 0.752 | Rec | 0.638(0.123-3.320) | 0.594 |
|  | AA | 2 | 8.7 |  | 6 | 13.0 | 0.599(0.111-3.242) | 0.552 | Add | 0.797(0.387-1.644) | 0.540 |
|  | rs1962142(C>T) |  |  |  |  |  |  |  |  |  |  |
|  | CC | 12 | 52.2 |  | 24 | 52.2 | 1.000 |  | Dom | 1.269(0.479-3.362) | 0.632 |
|  | CT | 11 | 47.8 |  | 18 | 39.1 | 1.773(0.615-5.110) | 0.289 | Rec | - | 0.989 |
|  | TT | 0 | 0.0 |  | 4 | 8.7 | - | 0.988 | Add | 0.952(0.427-2.121) | 0.905 |
|  | rs2001350(T>C) |  |  |  |  |  |  |  |  |  |  |
|  | TT | 14 | 60.9 |  | 22 | 47.8 | 1.000 |  | Dom | 0.643(0.229-1.808) | 0.402 |
|  | TC | 9 | 39.1 |  | 19 | 41.3 | 1.040(0.316-3.424) | 0.949 | Rec | - | 0.982 |
|  | CC | 0 | 0.0 |  | 5 | 10.9 | - | 0.982 | Add | 0.570(0.243-1.336) | 0.196 |
| *KEAP1* | rs1048290(G>C) |  |  |  |  |  |  |  |  |  |  |
|  | GG | 8 | 34.8 |  | 12 | 26.1 | 1.000 |  | Dom | 0.816(0.232-2.875) | 0.752 |
|  | GC | 12 | 52.2 |  | 19 | 41.3 | 1.093(0.283-4.218) | 0.898 | Rec | 0.368(0.094-1.439) | 0.151 |
|  | CC | 3 | 13.0 |  | 15 | 32.6 | 0.392(0.073-2.100) | 0.274 | Add | 0.633(0.286-1.397) | 0.257 |
|  | rs11545829(C>T) |  |  |  |  |  |  |  |  |  |  |
|  | CC | 12 | 52.2 |  | 20 | 43.5 | 1.000 |  | Dom | 0.778(0.284-2.136) | 0.627 |
|  | CT | 11 | 47.8 |  | 23 | 50.0 | 0.833(0.304-2.279) | 0.722 | Rec | - | 0.991 |
|  | TT | 0 | 0.0 |  | 3 | 6.5 | - | 0.991 | Add | 0.652(0.260-1.633) | 0.361 |
| *MAFF* | rs2267373(T>C) |  |  |  |  |  |  |  |  |  |  |
|  | TT | 10 | 43.5 |  | 14 | 30.4 | 1.000 |  | Dom | 0.434(0.137-1.378) | 0.157 |
|  | TC | 10 | 43.5 |  | 26 | 56.5 | 0.435(0.134-1.412) | 0.166 | Rec | 0.786(0.137-4.516) | 0.787 |
|  | CC | 3 | 13.0 |  | 6 | 13.1 | 0.430(0.061-3.035) | 0.398 | Add | 0.560(0.226-1.387) | 0.210 |
|  | rs4608623(G>T) |  |  |  |  |  |  |  |  |  |  |
|  | GG | 6 | 26.1 |  | 14 | 30.4 | 1.000 |  | Dom | 1.565(0.428-5.716) | 0.498 |
|  | GT | 10 | 43.5 |  | 17 | 37.0 | 1.838(0.413-8.185) | 0.425 | Rec | 0.983(0.314-3.076) | 0.976 |
|  | TT | 7 | 30.4 |  | 15 | 32.6 | 1.374(0.328-5.760) | 0.664 | Add | 1.134(0.570-2.254) | 0.720 |
|  | rs4444637(G>A) |  |  |  |  |  |  |  |  |  |  |
|  | GG | 20 | 87.0 |  | 34 | 73.9 | 1.000 |  | Dom | 0.309(0.072-1.320) | 0.113 |
|  | GA | 3 | 13.0 |  | 10 | 21.8 | 0.351(0.077-1.590) | 0.174 | Rec | - | 0.989 |
|  | AA | 0 | 0.0 |  | 2 | 4.3 | - | 0.989 | Add | 0.321(0.078-1.320) | 0.115 |
|  | rs4821767(A>C) |  |  |  |  |  |  |  |  |  |  |
|  | AA | 10 | 43.5 |  | 9 | 19.6 | 1.000 |  | Dom | 0.250(0.074-0.841) | 0.025 |
|  | AC | 10 | 43.5 |  | 27 | 58.7 | 0.276(0.081-0.945) | 0.040 | Rec | 0.397(0.081-1.960) | 0.257 |
|  | CC | 3 | 13.0 |  | 10 | 21.7 | 0.152(0.024-0.982) | 0.048 | Add | 0.353(0.142-0.878) | 0.025 |
| *MAFK* | rs4720833(G>A) |  |  |  |  |  |  |  |  |  |  |
|  | GG | 9 | 39.1 |  | 20 | 43.5 | 1.000 |  | Dom | 1.165(0.416-3.262) | 0.771 |
|  | GA | 14 | 60.9 |  | 20 | 43.5 | 1.536(0.527-4.473) | 0.431 | Rec | - | 0.980 |
|  | AA | 0 | 0.0 |  | 6 | 13.0 | - | 0.980 | Add | 0.814(0.367-1.807) | 0.613 |
|  | rs3808337(T>C) |  |  |  |  |  |  |  |  |  |  |
|  | TT | 7 | 30.4 |  | 19 | 41.3 | 1.000 |  | Dom | 1.620(0.534-4.911) | 0.394 |
|  | TC | 16 | 69.6 |  | 20 | 43.5 | 2.067(0.686-6.226) | 0.197 | Rec | - | 0.978 |
|  | CC | 0 | 0.0 |  | 7 | 15.2 | - | 0.979 | Add | 0.925(0.427-2.004) | 0.844 |

Abbreviations: ATLI, anti-tuberculosis drug-induced liver injury. Dom, dominant model; Rec, recessive model; Add, additive model.

^*^Conditional logistic regression model analysis and adjusted for weight and usage of hepatoprotectant.

**Supplementary Table 3** Genotypes distribution in ATLI cases and non-ATLI controls in **mixed** liver injury cases

| Gene | tagSNPs | ATLI Cases (N=40) | |  | non-ATLI controls (N=80) | | OR(95%CI)^*^ | *P* | Model | OR(95%CI)^*^ | *P* |
| --- | --- | --- | --- | --- | --- | --- | --- | --- | --- | --- | --- |
|  |  | N | % |  | N | % |  |  |  |  |  |
| *NRF2* | rs2886161(C>T) |  |  |  |  |  |  |  |  |  |  |
|  | CC | 11 | 27.5 |  | 28 | 35.0 | 1.000 |  | Dom | 1.387(0.573-3.355) | 0.468 |
|  | CT | 21 | 52.5 |  | 31 | 38.8 | 1.622(0.649-4.054) | 0.301 | Rec | 0.632(0.222-1.801) | 0.390 |
|  | TT | 8 | 20.0 |  | 21 | 26.2 | 0.865(0.256-2.915) | 0.815 | Add | 0.998(0.565-1.764) | 0.995 |
|  | rs4243387(T>C) |  |  |  |  |  |  |  |  |  |  |
|  | TT | 17 | 42.5 |  | 47 | 58.8 | 1.000 |  | Dom | 1.914(0.823-4.451) | 0.132 |
|  | TC | 19 | 47.5 |  | 24 | 30.0 | 2.009(0.844-4.782) | 0.115 | Rec | 0.952(0.244-3.710) | 0.943 |
|  | CC | 4 | 10.0 |  | 9 | 11.2 | 1.459(0.347-6.135) | 0.606 | Add | 1.436(0.768-2.686) | 0.257 |
|  | rs6726395(G>A) |  |  |  |  |  |  |  |  |  |  |
|  | GG | 13 | 32.5 |  | 32 | 40.0 | 1.000 |  | Dom | 1.469(0.604-3.572) | 0.397 |
|  | GA | 23 | 57.5 |  | 41 | 51.2 | 1.452(0.593-3.557) | 0.414 | Rec | 1.238(0.323-4.748) | 0.755 |
|  | AA | 4 | 10.0 |  | 7 | 8.8 | 1.674(0.363-7.717) | 0.509 | Add | 1.347(0.670-2.705) | 0.403 |
|  | rs1962142(C>T) |  |  |  |  |  |  |  |  |  |  |
|  | CC | 18 | 45.0 |  | 44 | 55.0 | 1.000 |  | Dom | 1.445(0.631-3.306) | 0.384 |
|  | CT | 20 | 50.0 |  | 30 | 37.5 | 1.576(0.669-3.716) | 0.298 | Rec | 0.694(0.123-3.907) | 0.679 |
|  | TT | 2 | 5.0 |  | 6 | 7.5 | 0.834(0.140-4.977) | 0.842 | Add | 1.196(0.619-2.311) | 0.594 |
|  | rs2001350(T>C) |  |  |  |  |  |  |  |  |  |  |
|  | TT | 18 | 45.0 |  | 50 | 62.5 | 1.000 |  | Dom | 2.361(0.946-5.896) | 0.066 |
|  | TC | 18 | 45.0 |  | 23 | 28.8 | 2.392(0.941-6.081) | 0.067 | Rec | 1.200(0.309-4.669) | 0.792 |
|  | CC | 4 | 10.0 |  | 7 | 8.7 | 2.174(0.480-9.835) | 0.313 | Add | 1.712(0.866-3.384) | 0.122 |
| *KEAP1* | rs1048290(G>C) |  |  |  |  |  |  |  |  |  |  |
|  | GG | 8 | 20.0 |  | 12 | 15.0 | 1.000 |  | Dom | 0.716(0.251-2.041) | 0.532 |
|  | GC | 26 | 65.0 |  | 52 | 65.0 | 0.771(0.265-2.240) | 0.632 | Rec | 0.585(0.164-2.088) | 0.409 |
|  | CC | 6 | 15.0 |  | 16 | 20.0 | 0.475(0.102-2.211) | 0.342 | Add | 0.701(0.333-1.477) | 0.350 |
|  | rs11545829(C>T) |  |  |  |  |  |  |  |  |  |  |
|  | CC | 18 | 45.0 |  | 34 | 42.5 | 1.000 |  | Dom | 0.830(0.362-1.902) | 0.659 |
|  | CT | 19 | 47.5 |  | 44 | 55.0 | 0.783(0.336-1.827) | 0.572 | Rec | 2.744(0.454-16.57) | 0.271 |
|  | TT | 3 | 7.5 |  | 2 | 2.5 | 2.281(0.339-15.36) | 0.397 | Add | 1.023(0.496-2.108) | 0.951 |
| *MAFF* | rs2267373(T>C) |  |  |  |  |  |  |  |  |  |  |
|  | TT | 16 | 40.0 |  | 30 | 37.5 | 1.000 |  | Dom | 0.938(0.428-2.056) | 0.873 |
|  | TC | 22 | 55.0 |  | 43 | 53.8 | 1.004(0.450-2.239) | 0.994 | Rec | 0.486(0.098-2.404) | 0.376 |
|  | CC | 2 | 5.0 |  | 7 | 8.7 | 0.487(0.090-2.628) | 0.403 | Add | 0.838(0.441-1.592) | 0.589 |
|  | rs4608623(G>T) |  |  |  |  |  |  |  |  |  |  |
|  | GG | 14 | 35.0 |  | 26 | 32.5 | 1.000 |  | Dom | 0.947(0.384-2.334) | 0.906 |
|  | GT | 20 | 50.0 |  | 44 | 55.0 | 0.895(0.355-2.259) | 0.815 | Rec | 1.351(0.438-4.167) | 0.601 |
|  | TT | 6 | 15.0 |  | 10 | 12.5 | 1.247(0.336-4.628) | 0.742 | Add | 1.073(0.560-2.054) | 0.832 |
|  | rs4444637(G>A) |  |  |  |  |  |  |  |  |  |  |
|  | GG | 32 | 80.0 |  | 67 | 83.8 | 1.000 |  | Dom | 1.416(0.528-3.793) | 0.489 |
|  | GA | 7 | 17.5 |  | 12 | 15.0 | 1.366(0.489-3.816) | 0.552 | Rec | 1.900(0.107-33.81) | 0.662 |
|  | AA | 1 | 2.5 |  | 1 | 1.2 | 2.027(0.109-37.72) | 0.636 | Add | 1.384(0.580-3.300) | 0.464 |
|  | rs4821767(A>C) |  |  |  |  |  |  |  |  |  |  |
|  | AA | 7 | 17.5 |  | 14 | 17.5 | 1.000 |  | Dom | 0.986(0.356-2.734) | 0.978 |
|  | AC | 25 | 62.5 |  | 50 | 62.5 | 1.017(0.353-2.933) | 0.975 | Rec | 0.898(0.351-2.301) | 0.823 |
|  | CC | 8 | 20.0 |  | 16 | 20.0 | 0.910(0.263-3.154) | 0.882 | Add | 0.950(0.513-1.758) | 0.870 |
| *MAFK* | rs4720833(G>A) |  |  |  |  |  |  |  |  |  |  |
|  | GG | 15 | 37.5 |  | 43 | 53.8 | 1.000 |  | Dom | 2.037(0.937-4.429) | 0.073 |
|  | GA | 17 | 42.5 |  | 32 | 40.0 | 1.622(0.705-3.731) | 0.255 | Rec | 4.127(1.054-16.16) | 0.042 |
|  | AA | 8 | 20.0 |  | 5 | 6.2 | 5.162(1.228-21.69) | 0.025 | Add | 2.000(1.096-3.650) | 0.024 |
|  | rs3808337(T>C) |  |  |  |  |  |  |  |  |  |  |
|  | TT | 17 | 42.5 |  | 41 | 51.3 | 1.000 |  | Dom | 1.476(0.670-3.248) | 0.334 |
|  | TC | 16 | 40.0 |  | 33 | 41.2 | 1.258(0.544-2.913) | 0.591 | Rec | 2.816(0.783-10.12) | 0.113 |
|  | CC | 7 | 17.5 |  | 6 | 7.5 | 3.203(0.814-12.60) | 0.096 | Add | 1.588(0.864-2.918) | 0.136 |

Abbreviations: ATLI, anti-tuberculosis drug-induced liver injury. Dom, dominant model; Rec, recessive model; Add, additive model.

^*^Conditional logistic regression model analysis and adjusted for weight and usage of hepatoprotectant.

**Supplementary Table 4** Genotypes distribution in ATLI cases and non-ATLI controls in **unclear** liver injury cases

| Gene | tagSNPs | ATLI Cases (N=101) | |  | non-ATLI controls (N=202) | | OR(95%CI)^*^ | *P* | Model | OR(95%CI)^*^ | *P* |
| --- | --- | --- | --- | --- | --- | --- | --- | --- | --- | --- | --- |
|  |  | N | % |  | N | % |  |  |  |  |  |
| *NRF2* | rs2886161(C>T) |  |  |  |  |  |  |  |  |  |  |
|  | CC | 40 | 39.6 |  | 72 | 35.6 | 1.000 |  | Dom | 0.818(0.475-1.408) | 0.468 |
|  | CT | 38 | 37.6 |  | 92 | 45.6 | 0.717(0.399-1.291) | 0.268 | Rec | 1.302(0.716-2.367) | 0.388 |
|  | TT | 23 | 22.8 |  | 38 | 18.8 | 1.068(0.535-2.133) | 0.852 | Add | 1.006(0.710-1.426) | 0.973 |
|  | rs4243387(T>C) |  |  |  |  |  |  |  |  |  |  |
|  | TT | 40 | 39.6 |  | 113 | 55.9 | 1.000 |  | Dom | 2.146(1.246-3.697) | 0.006 |
|  | TC | 54 | 53.5 |  | 72 | 35.6 | 2.322(1.323-4.075) | 0.003 | Rec | 0.855(0.332-2.203) | 0.746 |
|  | CC | 7 | 6.9 |  | 17 | 8.5 | 1.356(0.498-3.693) | 0.551 | Add | 1.530(1.018-2.301) | 0.410 |
|  | rs6726395(G>A) |  |  |  |  |  |  |  |  |  |  |
|  | GG | 34 | 33.6 |  | 82 | 40.6 | 1.000 |  | Dom | 1.422(0.828-2.443) | 0.202 |
|  | GA | 54 | 53.5 |  | 98 | 48.5 | 1.394(0.793-2.452) | 0.249 | Rec | 1.262(0.602-2.646) | 0.539 |
|  | AA | 13 | 12.9 |  | 22 | 10.9 | 1.534(0.680-3.464) | 0.303 | Add | 1.274(0.869-1.868) | 0.215 |
|  | rs1962142(C>T) |  |  |  |  |  |  |  |  |  |  |
|  | CC | 44 | 43.6 |  | 104 | 51.5 | 1.000 |  | Dom | 1.454(0.852-2.483) | 0.170 |
|  | CT | 51 | 50.5 |  | 84 | 41.6 | 1.592(0.904-2.804) | 0.108 | Rec | 0.817(0.304-2.198) | 0.689 |
|  | TT | 6 | 5.9 |  | 14 | 6.9 | 0.974(0.350-2.705) | 0.959 | Add | 1.201(0.801-1.800) | 0.376 |
|  | rs2001350(T>C) |  |  |  |  |  |  |  |  |  |  |
|  | TT | 46 | 45.5 |  | 116 | 57.4 | 1.000 |  | Dom | 1.560(0.962-2.530) | 0.072 |
|  | TC | 48 | 47.5 |  | 71 | 35.2 | 1.663(1.002-2.762) | 0.049 | Rec | 0.929(0.371-2.324) | 0.874 |
|  | CC | 7 | 7.0 |  | 15 | 7.4 | 1.129(0.437-2.915) | 0.803 | Add | 1.291(0.891-1.871) | 0.177 |
| *KEAP1* | rs1048290(G>C) |  |  |  |  |  |  |  |  |  |  |
|  | GG | 19 | 18.8 |  | 47 | 23.2 | 1.000 |  | Dom | 1.297(0.720-2.336) | 0.386 |
|  | GC | 63 | 62.4 |  | 111 | 55.0 | 1.396(0.757-2.576) | 0.286 | Rec | 0.847(0.460-1.558) | 0.593 |
|  | CC | 19 | 18.8 |  | 44 | 21.8 | 1.069(0.505-2.265) | 0.861 | Add | 1.043(0.725-1.499) | 0.821 |
|  | rs11545829(C>T) |  |  |  |  |  |  |  |  |  |  |
|  | CC | 41 | 40.6 |  | 79 | 39.1 | 1.000 |  | Dom | 0.928(0.547-1.574) | 0.782 |
|  | CT | 52 | 51.5 |  | 103 | 51.0 | 0.968(0.555-1.687) | 0.908 | Rec | 0.796(0.335-1.892) | 0.605 |
|  | TT | 8 | 7.9 |  | 20 | 9.9 | 0.783(0.316-1.939) | 0.597 | Add | 0.913(0.615-1.356) | 0.653 |
| *MAFF* | rs2267373(T>C) |  |  |  |  |  |  |  |  |  |  |
|  | TT | 52 | 51.5 |  | 82 | 40.6 | 1.000 |  | Dom | 0.639(0.387-1.056) | 0.080 |
|  | TC | 35 | 34.7 |  | 95 | 47.0 | 0.587(0.345-1.000) | 0.050 | Rec | 1.137(0.567-2.279) | 0.717 |
|  | CC | 14 | 13.8 |  | 25 | 12.4 | 0.854(0.401-1.817) | 0.682 | Add | 0.818(0.568-1.178) | 0.280 |
|  | rs4608623(G>T) |  |  |  |  |  |  |  |  |  |  |
|  | GG | 42 | 41.6 |  | 111 | 55.0 | 1.000 |  | Dom | 1.837(1.075-3.139) | 0.026 |
|  | GT | 31 | 30.7 |  | 55 | 27.2 | 1.564(0.846-2.891) | 0.153 | Rec | 1.923(1.027-3.601) | 0.041 |
|  | TT | 28 | 27.7 |  | 36 | 17.8 | 2.310(1.165-4.583) | 0.017 | Add | 1.525(1.089-2.135) | 0.014 |
|  | rs4444637(G>A) |  |  |  |  |  |  |  |  |  |  |
|  | GG | 79 | 78.2 |  | 166 | 82.2 | 1.000 |  | Dom | 1.261(0.703-2.262) | 0.436 |
|  | GA | 19 | 18.8 |  | 35 | 17.3 | 1.143(0.623-2.096) | 0.665 | Rec | 5.742(0.593-55.56) | 0.131 |
|  | AA | 3 | 3.0 |  | 1 | 0.5 | 5.946(0.612-57.81) | 0.124 | Add | 1.365(0.804-2.317) | 0.249 |
|  | rs4821767(A>C) |  |  |  |  |  |  |  |  |  |  |
|  | AA | 29 | 28.7 |  | 44 | 21.8 | 1.000 |  | Dom | 0.709(0.416-1.208) | 0.206 |
|  | AC | 44 | 43.6 |  | 96 | 47.5 | 0.703(0.392-1.261) | 0.237 | Rec | 0.890(0.530-1.495) | 0.660 |
|  | CC | 28 | 27.7 |  | 62 | 30.7 | 0.717(0.383-1.344) | 0.299 | Add | 0.849(0.618-1.168) | 0.315 |
| *MAFK* | rs4720833(G>A) |  |  |  |  |  |  |  |  |  |  |
|  | GG | 48 | 47.5 |  | 110 | 54.5 | 1.000 |  | Dom | 1.347(0.825-2.199) | 0.234 |
|  | GA | 45 | 44.6 |  | 73 | 36.1 | 1.445(0.865-2.413) | 0.159 | Rec | 0.797(0.317-2.002) | 0.629 |
|  | AA | 8 | 7.9 |  | 19 | 9.4 | 0.937(0.361-2.431) | 0.894 | Add | 1.150(0.788-1.677) | 0.469 |
|  | rs3808337(T>C) |  |  |  |  |  |  |  |  |  |  |
|  | TT | 46 | 45.5 |  | 108 | 53.5 | 1.000 |  | Dom | 1.398(0.855-2.284) | 0.181 |
|  | TC | 44 | 43.6 |  | 74 | 36.6 | 1.417(0.849-2.368) | 0.183 | Rec | 1.096(0.515-2.333) | 0.812 |
|  | CC | 11 | 10.9 |  | 20 | 9.9 | 1.318(0.589-2.948) | 0.502 | Add | 1.225(0.855-1.757) | 0.269 |

Abbreviations: ATLI, anti-tuberculosis drug-induced liver injury. Dom, dominant model; Rec, recessive model; Add, additive model.

^*^Conditional logistic regression model analysis and adjusted for weight and usage of hepatoprotectant.
